# Tracking Dabbing Using Search Query Surveillance: A Case Study in the United States

**DOI:** 10.2196/jmir.5802

**Published:** 2016-09-16

**Authors:** Zhu Zhang, Xiaolong Zheng, Daniel Dajun Zeng, Scott J Leischow

**Affiliations:** ^1^ Department of Management Information Systems The University of Arizona Tucson, AZ United States; ^2^ State Key Laboratory of Management and Control for Complex Systems Institute of Automation, Chinese Academy of Sciences Beijing China; ^3^ Department of Health Sciences Research Mayo Clinic Arizona Scottsdale, AZ United States

**Keywords:** marijuana, information seeking behavior, surveillance, search engine, time series analysis, spatial analysis

## Abstract

**Background:**

Dabbing is an emerging method of marijuana ingestion. However, little is known about dabbing owing to limited surveillance data on dabbing.

**Objective:**

The aim of the study was to analyze Google search data to assess the scope and breadth of information seeking on dabbing.

**Methods:**

Google Trends data about dabbing and related topics (eg, electronic nicotine delivery system [ENDS], also known as e-cigarettes) in the United States between January 2004 and December 2015 were collected by using relevant search terms such as “dab rig.” The correlation between dabbing (including topics: dab and hash oil) and ENDS (including topics: vaping and e-cigarette) searches, the regional distribution of dabbing searches, and the impact of cannabis legalization policies on geographical location in 2015 were analyzed.

**Results:**

Searches regarding dabbing increased in the United States over time, with 1,526,280 estimated searches during 2015. Searches for dab and vaping have very similar temporal patterns, where the Pearson correlation coefficient (PCC) is .992 (*P*<.001). Similar phenomena were also obtained in searches for hash oil and e-cigarette, in which the corresponding PCC is .931 (*P*<.001). Dabbing information was searched more in some western states than other regions. The average dabbing searches were significantly higher in the states with medical and recreational marijuana legalization than in the states with only medical marijuana legalization (*P*=.02) or the states without medical and recreational marijuana legalization (*P*=.01).

**Conclusions:**

Public interest in dabbing is increasing in the United States. There are close associations between dabbing and ENDS searches. The findings suggest greater popularity of dabs in the states that legalized medical and recreational marijuana use. This study proposes a novel and timely way of cannabis surveillance, and these findings can help enhance the understanding of the popularity of dabbing and provide insights for future research and informed policy making on dabbing.

## Introduction

**Figure 1:**
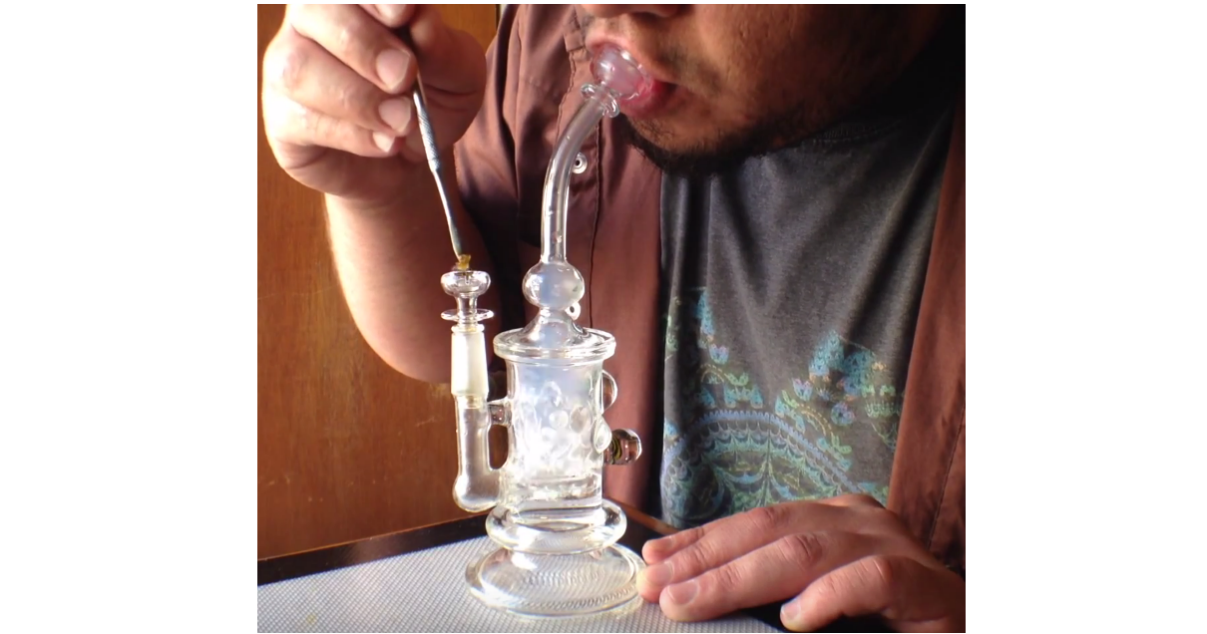
A YouTube video screenshot illustrating dabbing.

“Dabbing” is a colloquial term referring to the inhalation of vaporized marijuana concentrates and is an increasingly popular method of marijuana ingestion [1]. Marijuana concentrates contain high levels of delta-9-tetrahydrocannabinol (THC), which is the main psychoactive ingredient in marijuana. Butane hash oil (BHO), one of the major marijuana concentrates, is often produced by extracting THC from marijuana plants with liquid butane as the solvent. The resulting BHO products are often called “shatter,” “honeycomb,” “crumble wax,” “budder,” and “earwax” according to their form and quality [2]. Generally, a “dab” is used to describe a small amount of marijuana extract that is vaporized and inhaled using an “oil rig” (a specific dabbing device), vaporizer, or electronic nicotine delivery system (ENDS, also known as e-cigarettes) [3,4]. Figure 1 illustrates dabbing with a screenshot of a YouTube video.

Although dabbing can reduce the ingestion of smoke-related toxins and carcinogens that are typically inhaled when smoking cannabis, there are potential risks of dabbing that have not been studied sufficiently [5]. First, the high THC concentration and novel means of administration might result in some psychological and physical problems [1,4,6,7]. Second, researchers have found that burn injuries associated with BHO manufacture have increased in recent years [8,9]. Finally, concentrated cannabis extract for dabbing may be contaminated by residual solvent and pesticide during commercial or homemade production.

Existing studies about dabbing are scarce. The earliest study on dabbing found that the use of BHO had been outside the medical marijuana user community and it is viewed as significantly more dangerous compared with other forms of cannabis use [1]. Another study discovered that many baby boomers were exploring alternative cannabis products including cannabis concentrates to improve well-being and to reduce the potential risks of traditional marijuana smoking, as they got older and less healthy [10]. A recent paper investigated the contamination concerns of cannabis concentrates and cannabinoid transfer efficiency during dabbing [11]. Additionally, several studies investigated some problems associated with using ENDS to vape cannabis [5,12,13].

Because current national surveys in the United States do not track the use of marijuana extracts [4,14], some studies collected data on dabbing from social media, such as Twitter and YouTube, and obtained several significant findings. For instance, a study based on Twitter data suggested the popularity of dabs was greater in the states that legalized recreational and medical use or only medical use of cannabis [4]. Another study analyzed the content of 116 dabbing videos on YouTube and found that dabbing-related videos on YouTube can be easily accessed [14]. However, little is known about the temporal evolution and regional distribution of public perceptions and interest about dabbing, as well as the association between public interest in dabbing and the interest in ENDS across the United States.

Internet data such as Google searches have filled many public health data gaps [15-17], especially in behavioral outcomes, where traditional data such as telephone surveys are rare and expensive to generate [18]. Hundreds of studies exploited Google search data to yield valid insights in public health research [19]. For instance, Google search data have been used to estimate influenza epidemics [20-25], to track tobacco or emerging products such as ENDS [26-30], to study psychology-related problems [31-33], and to analyze cancer-related information seeking [34-36].

Given the great value of Google search data in digital surveillance systems for public health, this study aimed to fill some of the aforementioned knowledge gaps about dabbing using Google search data. In particular, this study characterizes (1) the popularity, on the Web, of dabbing across time compared with other forms of cannabis use; (2) the popularity of dabbing across the US states, including comparisons of searches across states with varying marijuana legalization policies; and (3) the correlations between dabbing-related searches and ENDS-related searches.

## Methods

### Data Collection

Data were collected from Google Trends [37], which provides a public and timely way to analyze the trends of certain search query terms by time, geographic location, and category. Each Google Trends curve consists of many weekly aggregated data points, and each data point indicates the fraction of searches for the chosen terms (or categories) in a geographic location at a particular time relative to the total number of searches at that time. Note that the value of each data point represents relative search volume (RSV). In a trend curve, all indices are scaled to the highest search week (RSV=100). Extremely low searches are normalized and scaled to be a zero volume (RSV=0). The study qualifies as nonhuman subjects research because this study does not involve data through intervention or interaction with a living individual or his or her identifiable private information.

**Figure 2:**
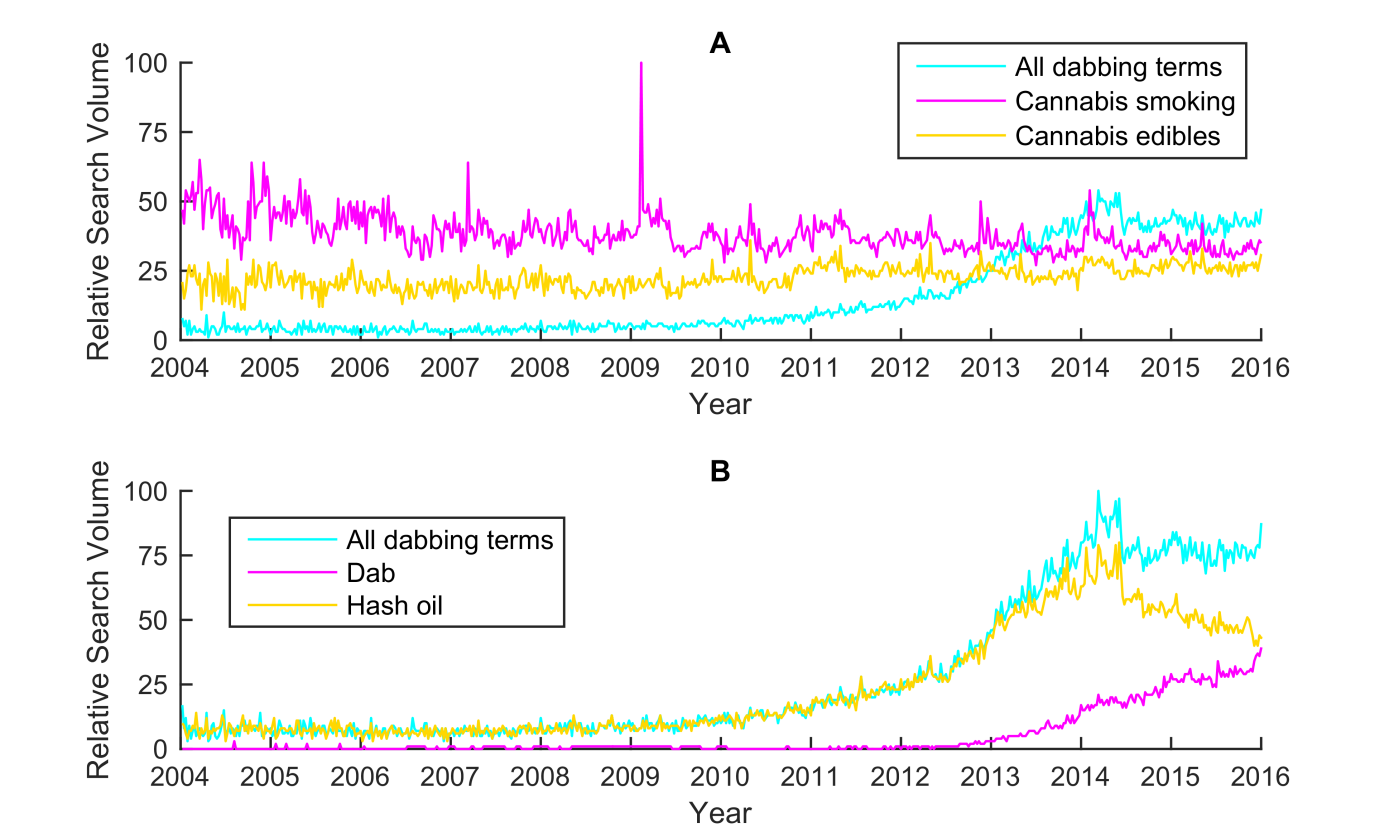
National trends for dabbing-related Google searches in the United States, 2004-2015. Panel A compared dabbing searches with searches for cannabis smoking and cannabis edibles, and panel B compared dabbing searches that included terms indicative of dab and hash oil.

**Figure 3:**
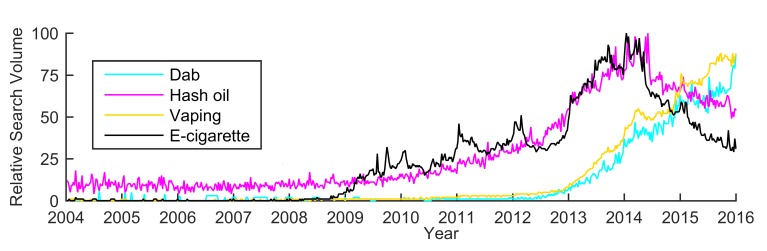
Temporal pattern comparison of searches for the dabbing and ENDS topics (ie, dab, hash oil, vaping, and e-cigarette). Each time series is on its own scale (ie, not applicable for relative search volume comparison between different time series).

**Figure 4:**
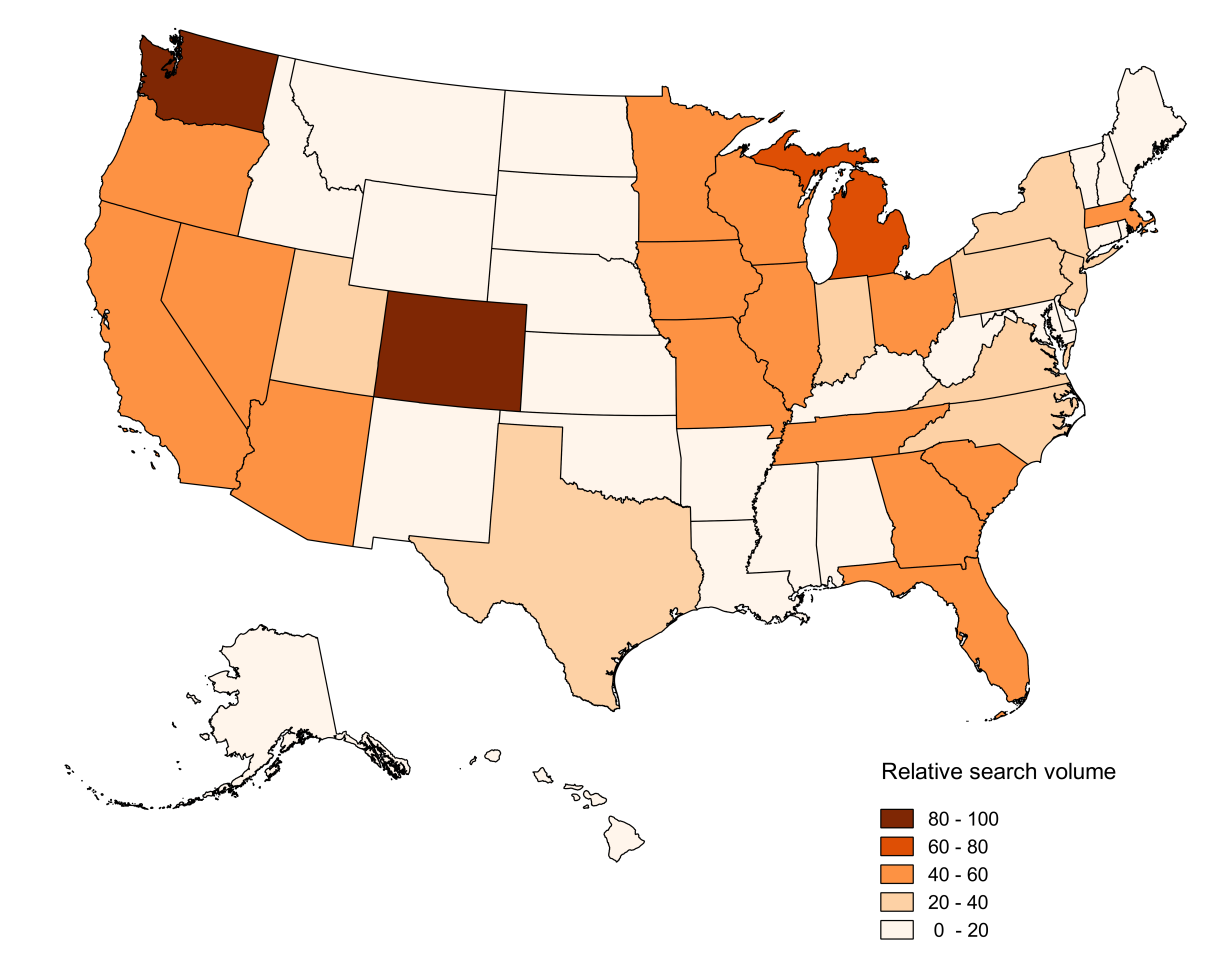
Choropleth map of raw searches regarding dabbing during 2015.

To understand the popularity of dabbing on the Web, the search topic “dabbing” was compared with the topics relating to other forms of cannabis use: “cannabis smoking” and “cannabis edibles” (see Figure 2) [38]. Note that all topics were on the same RSV scale. To examine the variations of dabbing search terms, “dabbing” was roughly divided into 2 smaller topics: “dab” and “hash oil” (see Figure 2). As a supplement to RSV data, Google AdWords [39] was used to estimate the raw search volume of the topic “dabbing” during 2015, which was similar to some previous studies exploiting advertisement keywords for infodemiology studies [30,40]. Furthermore, “dab” and “hash oil” were compared with 2 topics relating to ENDS (ie, “vaping” and “e-cigarette”) in terms of Google search queries (see Figure 3). The comparison was done to compare their individual temporal patterns, so each of the topics was on its own RSV scale. The division of ENDS into 2 topics was similar to that in a previous study [30], and the reason was also to examine the variations of search terms. Note that the time interval covered by the aforementioned data was between January 2004 (when data were first available) and December 2015. Finally, the search data relating to “dabbing” during 2015 were gathered to understand the popularity of dabbing across the US states (see Figure 4).

During data collection, basic query terms were initially identified according to related literature (eg, “dab rig,” “marijuana smoking,” “cannabis edibles”), and then related terms suggested by Google Trends were added to form candidate terms. Candidate terms were sorted by RSV, and terms with higher RSV were chosen because Google Trends limits the maximum number of words in query terms for a topic to 30. Unclear terms (eg, the single term “dab” can refer to the name of a bank) were omitted. The chosen terms were combined with “+” to form a composite term to collect the search data for a topic. For example, the composite term “dab rig+dab rigs+make dabs...” was used for the collection of searches relating to the topic “dabbing.” The specific terms used in data collection are provided in Multimedia Appendix 1.

### Data Analysis

The RSV of the topic “dabbing” for 2016 was predicted by using the autoregressive integrated moving average model and the R package called forecast [41]. To detect latent association between dabbing searches and ENDS searches, 2-tailed Pearson correlation coefficient (PCC) was adopted to analyze the pairwise correlations of the topics “dab,” “hash oil,” “vaping,” and “e-cigarette.”

The top 10 states with the highest RSV were obtained by sorting the raw search data relating to “dabbing” during 2015. The raw data for the 50 US states and the District of Columbia are provided in Multimedia Appendix 2. Differences in the 2015 raw dabbing data across US states with varying marijuana legalization policies were examined by 1-way ANOVA (analysis of variance) with 95% confidence interval. Similar to a prior study [4], states’ legal statuses before January 1, 2016, were grouped into 3 types: (1) type 1 includes 4 states and the District of Columbia that passed laws legalizing medical and recreational use of cannabis (Colorado, Washington, Alaska, Oregon, and District of Columbia); (2) type 2 includes 19 states that have legalized medical but not recreational use of cannabis (Arizona, California, Connecticut, Delaware, Hawaii, Illinois, Maryland, Maine, Massachusetts, Michigan, Minnesota, Montana, New York, Nevada, New Hampshire, New Jersey, New Mexico, Rhode Island, and Vermont); (3) type 3 includes 27 states that have not yet passed laws legalizing medical use of cannabis (Alabama, Arkansas, Florida, Georgia, Idaho, Indiana, Iowa, Kansas, Kentucky, Louisiana, Mississippi, Missouri, Nebraska, North Carolina, North Dakota, Ohio, Oklahoma, Pennsylvania, South Carolina, South Dakota, Tennessee, Texas, Utah, Virginia, West Virginia, Wisconsin, and Wyoming). Then the pairwise difference between the estimated group means was tested. Note that the time series plotting, correlation analysis, and ANOVA in this study were performed using MATLAB (Mathworks) [42].

## Results

As seen in panel A of Figure 2, searches relating to dabbing increased over time in the United States before 2014. The estimated dabbing searches during 2015 were 1,526,280. The predicted dabbing searches for 2016 were 22% (95% CI 19%-24%) more than dabbing searches during 2015. Before 2013, dabbing was less often searched than traditional cannabis smoking or cannabis edibles, but dabbing searches surpassed searches for cannabis smoking or cannabis edibles after the middle of 2013. For instance, searches regarding dabbing during 2015 were 28% (95% CI 25%-32%) more than searches regarding cannabis smoking and 58% (95% CI 54%-62%) more than searches regarding cannabis edibles.

As seen in panel B of Figure 2, hash oil searches occurred more often than searches for dab since 2004. Hash oil searches began increasing starting in 2008, and dab searches increased in the latter part of 2012. By the middle of 2014, searches regarding dab continued to increase, whereas searches for hash oil terms decreased. However, hash oil searches during 2015 were still 63% (95% CI 58%-68%) more than searches for dab.

In Figure 3, the search trends for hash oil and e-cigarettes show similar temporal patterns. Both had a wave peak in the early part of 2014. Similar results were also obtained between dab searches and vaping searches. The searches for dab and vaping showed a rapid increase beginning in 2013 and continued to show an increase.

Table 1 summarizes the temporal correlations between searches regarding dab or hash oil and searches regarding vaping or e-cigarettes. Searches for dab and hash oil have high correlations with searches of vaping and e-cigarette terms. For instance, the PCC for searches regarding dab and vaping is .992. The PCC for searches regarding hash oil and e-cigarettes is .931. Note that all *P* values for the abovementioned PCC values are less than .001.

**Table 1:** Temporal correlation of searches regarding dab and related topics. Note that the value in each cell is the Pearson correlation coefficient and all *P* values are less than .001.

Electronic nicotine delivery system	Dab	Hash oil
Vaping	.992	.783
E-cigarette	.600	.931

The 12 states with the highest raw RSV in 2015 were Colorado, Washington, Michigan, South Carolina, Nevada, Arizona, California, Oregon, Florida, Georgia, Missouri, and Massachusetts (see Figure 4). Note that Florida, Georgia, and Missouri had the same RSV. Among the Census Regions and Divisions of the United States, dabbing searches in the Pacific, East North Central, Middle Atlantic, and South Atlantic divisions were relatively higher than those in the other divisions.

**Figure 5:**
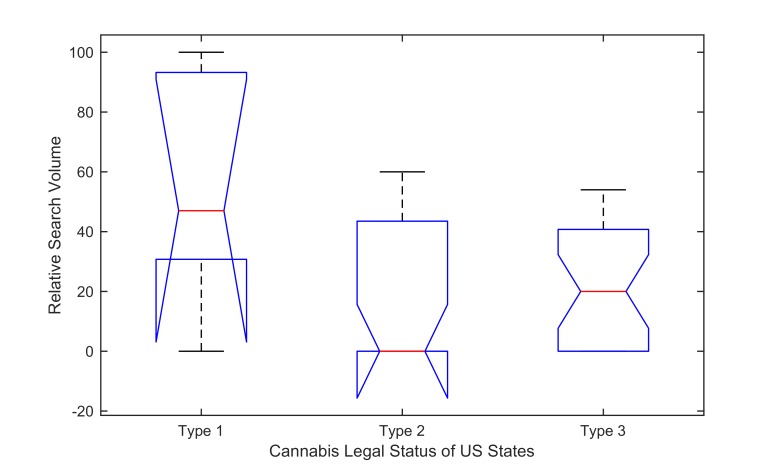
Raw dabbing Google searches by predictor for cannabis legal status of the United States. On each box, the central mark is the median, the edges of the box are the 25th and 75th percentiles, and the whiskers extend to the most extreme data points not considered outliers. Two medians are significantly different at the 5% significance level if their intervals of the notches do not overlap. Refer to the help document of [42] for more details of the box plots. Note that the details about the statuses are given in the Methods section.

During 2015, the means of searches for type 1, type 2, and type 3 were 55.800, 21.316, and 20.889, respectively. The group means of dabbing searches for type 1 and type 2 were significantly different at the 5% significance level (*P*=.02), and dabbing searches for type 1 and type 3 had similar results (*P*=.01). However, there was no significant difference at the 5% significance level between the group means of dabbing searches for type 2 and type 3 (*P*>.99; see Figure 5).

## Discussion

### Principal Findings

Dabbing searches are very common in the United States, and they have increased rapidly over time. Similar temporal patterns are found between searches for dab and vaping searches as well as searches for hash oil and e-cigarettes. Overall, dabbing was more frequently searched in the western states than other regions. The average dabbing searches were significantly higher in the states with recreational marijuana legalization than in the states without recreational marijuana legalization.

These findings fill some of the knowledge gaps regarding dabbing surveillance, but improved cannabis surveillance systems are needed to more fully understand the breadth and scope of variations in marijuana use. This study is the first to address the temporal associations between dabbing and ENDS. In general, the results based on search query monitoring can provide novel insights for further research and policy making.

Dabbing is becoming a popular alternative form of cannabis use. It became more popular than cannabis edibles and traditional cannabis smoking after the middle of 2013. When searches for dabbing are grouped into 2 categories, dab and hash oil, the searches demonstrate different developmental patterns over time. After 2014, searches for hash oil decreased, whereas dab searches increased. This suggests that the impact of the variations of dabbing-related terms used by cannabis users should be considered when designing survey questionnaires [30]. In addition, these temporal differences very likely reflect the changing technology opportunities afforded by the increased use of ENDS.

Previous studies have found that some dabbing users use ENDS for dabbing [5,12,13], which is a kind of emerging ENDS misuse. Its health risks and impact on cannabis control are still unknown. This study found that searches for dab and vaping searches have very similar temporal patterns, as do searches for hash oil and e-cigarettes. This finding suggests that there is a certain association between dabbing searches and ENDS searches. One possible reason is that a large number of people use ENDS for dabbing in the United States, but it still needs to be investigated further. In particular, searches for dab and vaping increased rapidly since 2013. Our results are almost consistent with the observations by a leading, popular marijuana magazine, High Times [43], which did a cover story on dabbing in July 2013. A senior editor stated that dabbing was an underground activity 5 months before the cover story [1].

On the basis of the editor’s claim, we can infer that the popularity of dabbing increased from an unobvious state before February 2013 to a relatively significant state in July 2013, which attracted the editor’s attention. However, the dabbing searches had begun to increase earlier than the time mentioned by the editor. One explanation is that the wisdom of crowds in Google Trends is more sensitive than individuals in terms of perceiving emerging phenomena, but the true reason still needs to be investigated. Considering the close associations between dabbing and ENDS, addressing dabbing issues together with ENDS may be an effective approach for a better understanding of how a variety of drugs are or can be delivered to the lungs using similar technology [5]. Further spatiotemporal analysis methods are needed to characterize their additional associations in the future [44,45].

Among the top 12 states with the largest raw RSV, all states legalized medical marijuana use except for South Carolina, Florida, Georgia, and Missouri. Some of these states (ie, Colorado, Washington, and Oregon) have already legalized recreational cannabis use. A recent study claims dabbing is more popular in states that have legalized medical marijuana use, which could be related to the emergence of vaporizer use among patients using medical marijuana and the recent increased availability of marijuana concentrates at medical marijuana dispensaries [4]. The claim was partially supported by the abovementioned results showing the temporal correlations between dabbing and ENDS.

This study found that dabbing searches are more prevalent on the West Coast of the United States, which is consistent with a prior study [4]. Previous analysis of Twitter data suggested that higher dabbing searches in western United States might be partially related to medical marijuana use laws that were passed much earlier there [4]. Another explanation was that the states on the West Coast have older and less strictly controlled medical marijuana programs. Besides, recreational marijuana legalization took effect in Washington after December 2012 and in Oregon after July 2015, so it is easier for people in these two states to do dabbing.

The average dabbing searches were significantly higher in the states with medical and recreational marijuana legalization than in the states with only medical marijuana legalization or the states without medical and recreational marijuana legalization. However, there was no significant difference in the means of dabbing searches between the states with only medical marijuana legalization and the states without medical and recreational marijuana legalization. The findings suggest greater popularity of dabs in the states that legalized medical and recreational marijuana use, which is partly consistent with the previous findings [4]. The comparison between this study and previous studies suggests that selecting suitable data and developing analytic standards are needed by those analyzing Web search data, social media, etc, just as those conducting surveillance and epidemiologic research have developed some standardized approaches.

Future research on dabbing and similar new technologies for drug delivery is needed to more fully understand use patterns that are tied to demographic characteristics, policy changes, drug availability, changes in technology, and other variables. In addition, there is a need to assess whether searches on topics such as dabbing are associated with actual use patterns (eg, as determined by sales patterns) and reports of adverse events (eg, via poison control or Food and Drug Administration reporting). If clear temporal relationships can be demonstrated between dabbing search changes over time and specific measurable behaviors or health outcomes, it will be possible to more fully characterize the public health value of tracking dabbing and other similar search outcomes as an early warning system of emerging substance use and abuse.

### Limitations

Some limitations need to be taken into account when interpreting this study. First, the Google Trends data in this study are the adjusted relative search values, because Google Trends does not provide actual search volume data. Data for only popular terms are analyzed by Google Trends, which causes the RSV of search terms with extremely low volume to appear as 0. Google Trends data lack demographic information compared with survey data. Although Google Trends data include only search activities using Google, Google accounts for an estimated 65% of the market share of search engines in the United States between January 2008 and October 2015 [46]. Second, the data in this study are limited to the United States and English search terms, so caution is needed when generalizing our results to other countries. The selection of search term keywords might lead to some minor biases; however, nearly all of our search term keywords have almost reached the number limit of Google Trends search terms. Third, the search data have not been assessed relative to year-by-year trends in actual use of products, so we cannot at this time suggest that search trends on dabbing predicted eventual increases in actual use of those products, but future research will assess those relationships. Finally, our results demonstrate high correlations between dabbing and ENDS in terms of Google searches. The reasons for the positive correlations between dabbing and ENDS searches require additional research across a variety of disciplines, although we provided some possible explanations.

### Conclusions

In recent years, the general public has increasingly accepted marijuana legalization, but the potential adverse effects and health risks of dabbing are still being researched. This study provides a novel and timely way of conducting cannabis-related surveillance that may complement the current but limited epidemiologic data on dabbing. In the future, fusing Web-based data, such as Google searches and Web-based surveys, and offline community-recruited samples may help enhance the understanding of dabbing and similar substance use and provide insights for relevant research and informed policy making.

## Appendix

Multimedia Appendix 1 The search terms for collecting Google Trends data.

Multimedia Appendix 2 The raw Google searches for the 50 US states and the District of Columbia.

## Abbreviations

ANOVA: analysis of variance
BHO: butane hash oil
ENDS: electronic nicotine delivery system
PCC: Pearson correlation coefficient
RSV: relative search volume
THC: delta-9-tetrahydrocannabinol
