# Supplementary material for: Tracking Dabbing Using Search Query Surveillance: A Case Study in the United States
Source: J Med Internet Res. 2016 Sep 16;18(9):e252. doi: 10.2196/jmir.5802 (PMC5045525; doi:10.2196/jmir.5802)
Supplement: Multimedia Appendix 1 [file jmir_v18i9e252_app1.pdf]

Multimedia Appendix 1: The Search Terms for Collecting Google Trends Data

**Dabbing**		**Cannabis smoking**	**Cannabis edibles**	**ENDS**	
**Dab**	**Hash oil**			**Vaping**	**E-cigarette**
dab rig<br>dab rigs<br>make dabs<br>dab nail<br>dab glass<br>smoke dabs<br>make dab	hash oil<br>marijuana oil<br>thc oil<br>hash wax<br>marijuana wax<br>bho oil<br>make bho	smoking marijuana<br>smoke marijuana<br>marijuana pipes<br>marijuana pipe<br>marijuana bong<br>marijuana joint<br>marijuana bongs<br>marijuana bowl<br>smoke cannabis<br>smoking cannabis<br>marijuana blunt<br>marijuana bowls<br>smokes marijuana<br>marijuana joints	cannabutter<br>marijuana tea<br>marijuana brownies<br>marijuana recipe<br>marijuana butter<br>marijuana recipes<br>marijuana food<br>bhang<br>drinking marijuana<br>eating marijuana<br>drink marijuana<br>marijuana cooking<br>cannabis butter<br>eat marijuana	vape<br>vaping<br>vapes<br>vaporizer pen<br>vaper<br>vapor pen<br>vapers	e cig<br>electronic cigarette<br>e cigarette<br>ecig<br>electronic cigarettes<br>e cigs<br>e cigarettes
